# Gene expression profiles for low-dose exposure to diethyl phthalate in rodents and humans: a translational study with implications for breast carcinogenesis

**DOI:** 10.1038/s41598-020-63904-w

**Published:** 2020-04-27

**Authors:** Kalpana Gopalakrishnan, Vasily N. Aushev, Fabiana Manservisi, Laura Falcioni, Simona Panzacchi, Fiorella Belpoggi, Humberto Parada, Gail Garbowski, Hanina Hibshoosh, Regina M. Santella, Marilie D. Gammon, Susan L. Teitelbaum, Jia Chen

**Affiliations:** 1 Department of Environmental Medicine and Public Health, Icahn School of Medicine at Mount Sinai, New York, NY USA; 2 Cesare Maltoni Cancer Research Centre, Ramazzini Institute, Bentivoglio, Bologna Italy; 3 Graduate School of Public Health, San Diego State University, San Diego, CA USA; 4 Department of Environmental Health Sciences, Columbia University, New York, NY USA; 5 Department of Pathology and Cell Biology, Columbia University Medical Center, New York, NY USA; 6 Department of Epidemiology, University of North Carolina at Chapel Hill, Chapel Hill, NC USA; 7 Department of Pediatrics, Icahn School of Medicine at Mount Sinai, New York, NY USA; 8 Department of Medicine, Hematology and Medical Oncology, Icahn School of Medicine at Mount Sinai, New York, NY USA; 9 Department of Oncological Sciences, Icahn School of Medicine at Mount Sinai, New York, NY USA

**Keywords:** Breast cancer, Breast cancer

## Abstract

Phthalates are commonly included as ingredients in personal care products such as cosmetics, shampoos and perfumes. Diethyl phthalate (DEP) has been found to be anti-androgenic and linked with adverse reproductive effects on males, but effects on females are poorly understood. We designed an integrative and translational study to experimentally examine the effects of DEP exposure at a human-equivalent dose on the mammary transcriptome in rats and to subsequently examine the DEP gene signature in breast tissues (both pre-malignant and tumor) from a population study. In Sprague-Dawley rats treated orally with DEP from birth to adulthood, we identified a signature panel of 107 genes predominantly down-regulated by DEP exposure. Univariate analysis of this 107 DEP gene signature in pre-malignant breast tissues revealed that six genes (*P4HA1*, *MPZL3*, *TMC4*, *PLEKHA6*, *CA8*, *AREG*) were inversely associated with monoethyl phthalate (MEP; the urinary metabolite of DEP) concentration (p < 0.05) among postmenopausal women; all six genes loaded on to one of seven factors identified by factor analysis. Transcription factor enrichment analysis revealed that genes in this factor were enriched for androgen receptor binding sites. These six genes were also significantly down-regulated in pre-malignant adjacent tissues compared to the corresponding tumor tissues in pair-wise analyses (p < 0.05). Results from our translational study indicate that low level exposure to diethyl phthalate results in measurable genomic changes in breast tissue with implications in breast carcinogenesis.

## Introduction

Phthalates are a family of chemicals with diverse properties that are added to a wide variety of consumer products and used in industrial and medical applications^1^. One of the congeners, diethyl phthalate (DEP), is commonly used as a plasticizer and solvent in personal care products such as cosmetics, shampoos and perfumes and in pharmaceutical products^1^. The United States (U.S.) Food and Drug Administration found DEP to be the most common phthalate in personal care products^2^, a major source of exposure to DEP^3^. The urinary metabolite of DEP, monoethyl phthalate (MEP), is detectable in U.S. population samples at levels that are often an order of magnitude higher than other phthalates such as di(2-ethylhexyl) phthalate and dibutyl phthalate^4^. Due to non-covalent bonding to its parent materials, DEP is leached easily into the environment resulting in widespread human exposure^1^.

DEP has been shown to have anti-androgenic effects in males, including shorter anogenital distance^5^ and increased sperm DNA damage^6,7^. MEP has been detected in breast milk, suggesting lactational exposure, and was associated with lowered testosterone in infant males^8^. Links between phthalate exposures and female developmental and reproductive and cancer outcomes, however, have been less well studied, despite their potential as endocrine disruptors. Reduced fecundity with MEP exposure was reported in a Danish cohort^9^. An epidemiologic study in Mexican women showed an association between MEP exposure and increased breast cancer risk^10^. High serum level of MEP in postmenopausal women was associated with elevated breast density^11^, a marker for breast cancer risk^12^. We recently reported results from the Long Island Breast Cancer Study Project (LIBCSP) in which we did not observe any significant associations between urinary level of MEP and breast cancer risk, while inverse associations were observed for mono(3-carboxypropyl) phthalate (MCPP) and monocarboxyoctyl phthalate (MCOP), metabolites of anti-androgenic phthalates^13^.

Breast cancer is considered a hormone-driven disease^14^. Given the hormone-disrupting properties of DEP and its widespread exposure through the use of personal care and consumer products, there is an urgent need to systematically study whether DEP exposures may impact breast cancer development. In rats, fetal and prepubertal exposures to high doses of butyl benzyl phthalate led to changes in gene expression profiles of mammary glands^15,16^. Less is known about the effects of DEP exposure particularly at doses that are relevant to human exposure.

The aim of our present study is to employ an animal model to experimentally identify a gene signature of DEP exposure at a human-equivalent dose and to subsequently translate these findings to humans. We exposed female Sprague-Dawley (SD) rats to DEP orally from birth to adulthood at a dose that was previously shown to produce a urinary metabolite (MEP) level similar to that of the U.S. population^17^. We identified a DEP gene signature in normal developing mammary glands in SD rats, and subsequently examined this signature in pre-malignant and breast cancer tissues from a subsample of women who participated in a population-based study. The overall goal of the study is to examine whether human level exposure to DEP induces measurable transcriptomic changes in target tissues, thus shedding light on the possible causal relationship between phthalate exposure and breast cancer.

## Materials and Methods

### Test compound

Diethyl phthalate (DEP) (CAS # 84-66-2, lot # STBB0862V, 99% purity) was supplied in plastic containers (Sigma Aldrich, Italy). Olive oil, supplied in glass bottles (Montalbano Agricola Alimentare Toscana, Florence, lot # 111275, Italy), was used as the vehicle to prepare the dosing solution. The experimental oral dose was 0.1735 mg/Kg/day, which represented 1/10,000 the no observed adverse effect levels (NOAEL) of DEP^18–20^. DEP was stored in the dark at room temperature (20 °C). The solutions were prepared weekly on the basis of mean body weight of each group and were continuously stirred before and during the treatment. To minimize external contamination, olive oil and DEP were stored in glass containers and administered using 5 mL glass syringes. Biological samples were collected in polypropylene vials. Chemical analyses and stability testing have been described previously^21^.

### Animal studies

Animal studies were carried out at Cesare Maltoni Cancer Research Centre/Ramazzini Institute (CMCRC/RI) (Bentivoglio, Italy) in accordance with the rules of Italian law for Animal Welfare (Decreto Legislativo 26, 2014), following the principles of Good Laboratory Practices and Standard Operating Procedures of the CMCRC/RI facility, which include authorization by the ethical committee. At Mount Sinai, the study was approved by the Institutional Animal Care and Use Committee (IACUC). The experiment used female Sprague-Dawley (SD) rats which belong to the colony that has been used for over 40 years in the laboratory of the CMCRC/RI. There were no siblings in each treatment group and they were randomized so as to have minimal differences in body weight among them (standard deviation <10% of the average). Animals were housed in makrolon cages (41 × 25 × 15 cm) at two or three per cage, with a stainless steel wire top and a shallow layer of white wood shavings as bedding (Giuseppe Bordignon supplier, Treviso, Italy). All animals were kept in a single room at 23 ± 3 °C and 40–60% relative humidity. Lighting was artificial and the light/dark cycles were approximately 12 hours each. All animals were given the same standard “Corticella” pellet diet (Piccioni Laboratory, Milan, Italy). Feed and tap water were available *ad libitum* and were both periodically analyzed to exclude biological and chemical contamination (mycotoxins, pesticides, arsenic, lead, mercury, selenium).

There were four experimental groups: parous and age-matched nulliparous, treated with DEP or vehicle, olive oil. There were five animals per experimental group. Animals were weighed weekly to determine treatment dose. F0 generation corresponds to breeders of the experimental animals (F1). F1 animals were treated daily from postnatal day (PND) one to PND 28 through milk of their dams (F0), which were gavaged with DEP or vehicle. After weaning at PND 28, F1 animals were treated with DEP or vehicle by gavage three times a week until PND 180 and sacrificed at PND 181. Parous rats were mated (outbred) at PND 97 and treatment was continued through pregnancy, delivery of pups (F2) and lactation. At the time of sacrifice of parous rats on PND 181, F2 pups had completed weaning. Animals were euthanized via carbon dioxide inhalation and necropsy was immediately performed for collection of mammary tissues. Animals were sacrificed on the same day and at random during the estrous cycle.

### Transcriptome profiling in rats

The fifth left and right caudal mammary glands were collected, pulverized in liquid nitrogen and total RNA was extracted using Maxwell 16 LEV simplyRNA Blood kit (Promega, WI) or by Direct-zol RNA MiniPrep kit (Zymo Research, CA). RNA concentration was determined using NanoDrop (Thermo Scientific, MA) and RNA quality was assessed using a 2100 Bioanalyzer (Agilent Technologies, CA); samples with RNA Integrity Number ≥ 7 were used for microarrays. Transcriptomes were profiled by GeneChip Rat Gene 2.0 ST arrays (Affymetrix, CA) at the Yale Center for Genome Analysis (Yale School of Medicine, CT) as described previously^22^. Quality control of .CEL files and pre-processing by robust multiarray average (RMA) method were done using expression console software (Affymetrix, CA). Batch effects due to RNA extraction method were removed using ComBat package^23^ in RStudio (R 3.0.2). We applied a signal intensity filter to retain only those probesets with high and stable expression (signal value >30th percentile in at least one experimental group). A variance-based filter was used to retain the top 50% of probesets with high interquartile range resulting in a final dataset containing 7,532 genes. Microarray data have been deposited in NCBI’s Gene Expression Omnibus (GEO) and are accessible through GEO Series accession number GSE95554 (https://www.ncbi.nlm.nih.gov/geo/query/acc.cgi?acc=GSE95554)

### Study population

We utilized resources from the Long Island Breast Cancer Study Project (LIBCSP), which includes a population-based sample of women newly diagnosed with first primary breast cancer who participated in the main study interview within two to three months of diagnosis^24^. Approval of the Institutional Review Board (Program for the Protection of Human Subjects) was obtained by all participating institutions and informed consent was obtained from all study participants. Study participants included 1,508 women diagnosed with first primary *in situ* or invasive breast cancer between 1996 and 1997 who resided in Nassau and Suffolk counties on Long Island, NY. At the time of the in-person baseline interview, 93% of participants donated 25 mL spot urine samples, which were shipped overnight on ice, processed, and banked at −20 °C. Archived tumor tissue of the first primary breast cancer was obtained from the diagnosing hospitals^25^. Demographic, reproductive and lifestyle characteristics of women with available pre-malignant adjacent tissue and urinary MEP concentrations (n = 294), are provided in Table 1.

**Table 1:** Participant characteristics.

n	Subset	Full
	294	892
Age at diagnosis: mean (SD)	57.9 (12.7)	59.3 (12.8)
Stage = invasive (%)	262 (89.1)	768 (86.1)
Race = white (%)	274 (93.2)	835 (93.6)
Menopausal status = postmenopausal (%)	189 (65.6)	605 (67.8)
Parity = 1 or more (%)	254 (86.4)	769 (86.2)
Lactated (%)	96 (32.7)	292 (32.7)
Age at menarche: mean (SD)	12.6 (1.6)	12.5 (1.6)
Age at first birth: mean (SD)	25.7 (4.8)	25.7 (4.9)
Oral contraceptive use (%)	141 (48.0)	377 (42.3)
Hormone replacement therapy use (%)	82 (28.0)	246 (27.6)
Current or past smoker (%)	165 (56.1)	485 (54.3)
Alcohol (%)	180 (61.2)	556 (62.3)
BMI: mean (SD)	26.9 (6.1)	26.9 (5.8)
Education:		
≤High School	142 (48.5)	443 (49.8)
College	112 (38.2)	316 (35.5)
Post-college	39 (13.3)	131 (14.7)
Income:		
≤$24,999	50 (19.5)	175 (22.2)
$25,000-$49,999	90 (35)	237 (30.2)
≥$50,000	117 (45.5)	374 (47.6)

All LIBCSP women with urinary MEP measurements (n = 892) compared with subset with both urinary MEP measurement and DEP gene signature measurement in pre-malignant adjacent tissues (n = 294).

Tumor tissue was excised prior to treatment initiation. Formalin-fixed, paraffin-embedded (FFPE) tumor sections were histopathologically reviewed by a trained pathologist and the cancer tissue was separated using manual microdissection for 745 women, and histologically normal pre-malignant adjacent tissue was retrieved for 586 of them. Following RNA extraction and quality control, samples were cross-referenced with DEP exposure information to obtain the final number of samples used in this study: 294 pre-malignant adjacent tissues, of which 243 had a paired tumor tissue.

Monoethyl phthalate (MEP) was measured in spot urine samples donated by 892 women with breast cancer using standard laboratory techniques by staff at the Centers for Disease Control and Prevention. Briefly, metabolites were deconjugated enzymatically, matrix removal and analyte enrichment were accomplished by solid phase extraction, and instrumental analysis was done with high performance liquid chromatography–tandem mass spectrometry using isotope dilution quantification as described before^26^.

### Gene expression profiling in LIBCSP

Genes found to be differentially expressed by DEP exposure in rat mammary tissues were converted to orthologous human IDs using the Gene Annotator tool in the rat genome database^27^, resulting in a panel of 107 genes. These genes were included as part of a custom-designed code-set (NanoString Technologies, WA) to determine expression of the DEP gene signature in breast tumor and pre-malignant adjacent tissues of the LIBCSP. Total RNA was extracted from FFPE breast tumor and adjacent tissues using the Qiagen miRNeasy FFPE kits (Qiagen, MD). RNA concentration and quality were determined using NanoDrop (Thermo Scientific, MA). Only those samples exceeding concentration of 10 ng/µL were used. 100 ng RNA was incubated with reporter and capture probes overnight at 65 °C. Following hybridization, unbound probes were removed, and the purified complexes were aligned and immobilized on imaging cartridges using an nCounter Prep station. Code count detection was carried out by scanning cartridges in an nCounter Digital Analyzer to determine gene expression levels. Raw nCounter code counts were first normalized against the geometric mean of spike-in control probes to minimize the impact of sample preparation and detection. For background determination, counts that fell below two standard deviations of the mean of negative control probes were deemed unexpressed and assigned a value equivalent to the background threshold divided by the square root of two. Finally, data were normalized against the geometric mean of 6 housekeeping genes with high and stable expression: *CLTC*, *GAPDH*, *GUSB*, *HPRT1*, *PGK1* and *TUBB*. The final dataset was log_2_ transformed.

### Statistical methods

#### Differential gene expression analysis in rats

Differential gene expression analysis between DEP and control in rats was performed using linear models for microarray data (limma) package^28^. To obtain a robust DEP gene signature, we bootstrapped samples by choosing four out of five samples in treatment groups and in control groups resulting in 25 rounds of differential gene expression analysis each for parous and for nulliparous groups. A lenient false discovery rate (FDR) of 25% using Benjamini-Hochberg (BH) correction^29^ and a fold change of ≥1.5 fold was used. For functional enrichment analysis of differentially expressed genes we used gene ontology (GO)^30^ via EnrichR^31^. Fisher’s exact test^32^ was used to assess significance of enrichment at a FDR of 5% by BH method.

#### Signature gene analysis in the LIBCSP

Gene expression data of pre-malignant adjacent tissues as well as urinary MEP concentration were available for 294 breast cancer cases. Examination of the association between urinary MEP concentration and expression of each gene in the DEP gene signature was carried out using generalized linear models^33^ before and after stratifying by menopausal status. Natural log transformed urinary MEP concentration was quartiled, and the median of each quartile was considered as the predictor. To reduce the dimensionality of the dataset, we performed factor analysis to group highly correlated genes into latent factors using the psych package^34^ with a correlation cutoff of 0.3 to load a gene into a factor. We subsequently carried out an examination of the association between quartiles of urinary MEP concentration with the latent factors, such as oral contraceptive use, hormone replacement therapy, lactation, smoking status, religion etc. Differential gene expression analysis between pre-malignant adjacent and corresponding tumors in this study (n = 243) as well as in the breast cancer TCGA microarray data (n = 61 tumors and adjacent samples) filtered to include only the DEP gene signature, was carried out using Wilcoxon signed rank test^35^. To gain insight about upstream molecular regulators of genes contained within factors, we performed enrichment for transcription factors using ‘ChEA 2016’ via Enrichr^31^ which uses Fisher’s exact test^32^. Multiple comparisons were adjusted using BH-based FDR of 5%.

A full analysis of the association between phthalate exposure and breast cancer incidence in the LIBCSP cases and population-based controls has been reported previously^13^.

### Ethics approval and consent to participate

Animal studies were performed following the principles of Good Laboratory Practices and Standard Operating Procedures of the CMCRC/RI facility, which include authorization by the ethical committee; the study was approved by the Institutional Animal Care and Use Committee (IACUC) of Mount Sinai. For the Long Island Breast Cancer Study Project (LIBCSP), Institutional Review Board approval was obtained by all participating institutions and informed consent was obtained from all study participants; study was approved by the Institutional Review Board of Mount Sinai (Program for the Protection of Human Subjects). All experiments were performed in accordance with relevant guidelines and regulations.

## Results

### Identifying a DEP gene signature in rats

**Figure 1:**
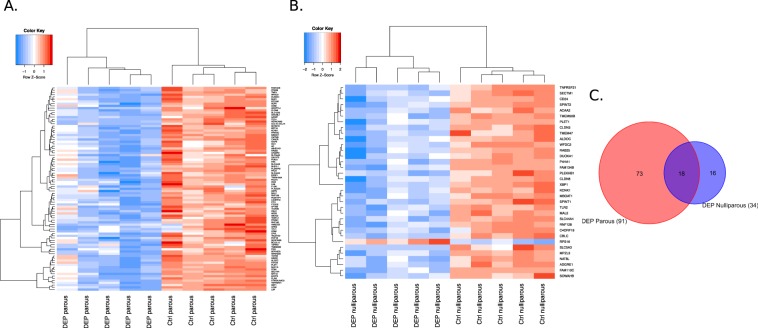
DEP gene signatures in adult (**A**) parous and (**B**) age-matched nulliparous Sprague-Dawley rats identified by limma analysis of bootstrapped samples (FDR < 0.25, fold change ≥1.5). (**C**) Overlap of parous and nulliparous gene signatures. A full list of gene names in each group is provided in Supplemental Information (Table S1).

Our final dataset of ~7,500 genes was obtained after filtering the mammary microarray data to retain only those genes with both high expression and variation. We then performed differential gene expression analysis between DEP-treated rats and controls to identify a phthalate gene signature. To identify a robust signature, we bootstrapped samples in parous and nulliparous rats separately given the potential difference in mammary transcriptome. This resulted in 339 and 43 differentially expressed genes by DEP treatment in the parous and nulliparous rat groups, respectively. For the parous rat group, we ranked genes by their difference in median expression between treatment and control and selected the top 100 genes. Finally, we identified the human orthologs of the rat genes resulting in 91 orthologous human genes in the parous group (‘DEP parous gene signature’) and 34 orthologous human genes in the nulliparous group (‘DEP nulliparous gene signature’) (Supplementary Table 1). These gene signatures separated DEP-treated from the control groups (Fig. 1A,B). All genes in both parous and nulliparous signatures were down-regulated by DEP exposure, except for *Rps16* which was up-regulated in the nulliparous rat group. Eighteen genes overlapped between the parous and nulliparous signature, resulting in a final gene panel of 107 genes (‘DEP gene signature’) (Fig. 1C). Pathway enrichment analysis of these 107 genes revealed enrichment of gene ontology (GO) terms ‘mammary gland alveolus development’ (*TPH1*, *AREG*, *PRLR*), ‘response to hypoxia’ (*MUC1*, *ACAA2*, *CLDN3*, *TGFB3*, *ALDOC*, *ANGPTL4*, *CD24*, *TLR2*), ‘response to steroid hormone’ (*FOXA1*, *SLC34A2*, *AR*, *KRT19*, *TGFB3*, *PTGDS*, *CD24*, *AREG*, *TLR2*, *ABCG2*) and ‘cellular amine metabolic process’ (*SLC44A3*, *TPH1*, *SLC44A4*, *AADAT*, *PLA2G2A*, *MBOAT1*) (FDR < 0.05).

### DEP gene signature in humans

**Figure 2:**
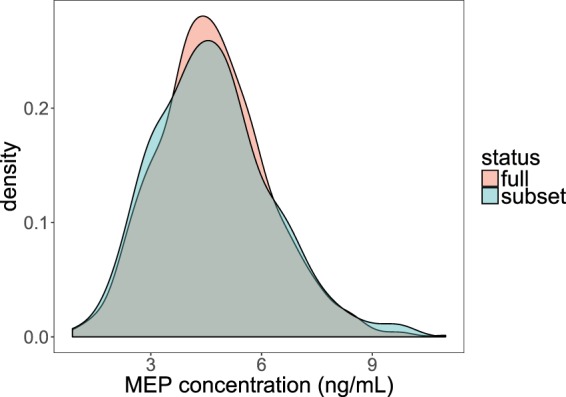
Comparative density distributions of monoethyl phthalate (MEP) concentrations between all samples with urinary MEP concentration (n = 892) and subset of samples with both MEP concentration and DEP gene signature measurement in pre-malignant adjacent breast tissues (n = 294) among LIBCSP case women.

Among the LIBCSP case cohort, we have 294 subjects with both gene profile and urinary MEP measurement. We compared the full group of cases with urinary MEP information (n = 892) with the subset (n = 294) used in our investigation and found no major differences in clinical, demographic and reproductive factors (Table 1), nor did we detect any difference in MEP distribution between the two groups (Fig. 2).

**Figure 3:**
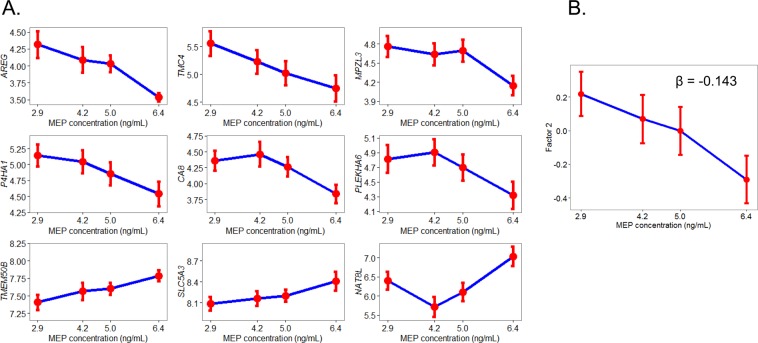
(**A**) Association between DEP gene signature and MEP concentration in pre-malignant adjacent breast tissues among postmenopausal women (n = 189; p < 0.05). (**B**) Association between factor 2 and MEP concentration (p < 0.05). Points on graph depict mean +/− standard error.

**Figure 4:**
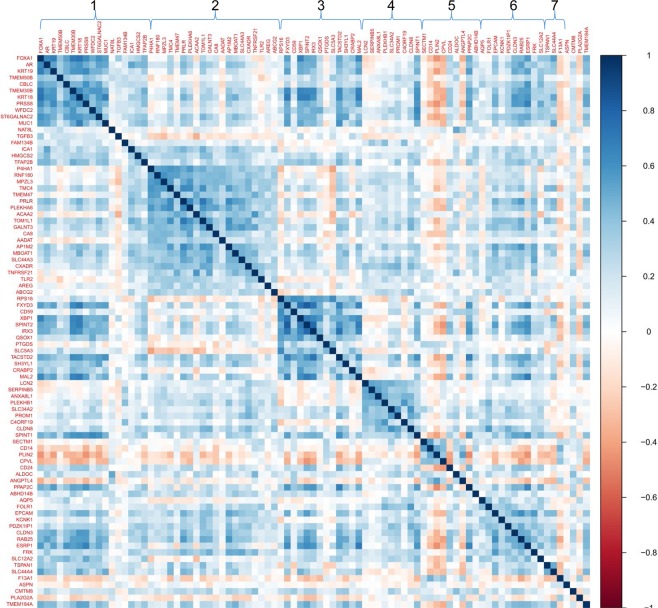
Correlation structure of DEP gene signature in pre-malignant adjacent breast tissues among LIBCSP postmenopausal women, where genes are ordered by factors as indicated by numbers on top of figure.

We examined the expression of the 107 DEP gene signature in pre-malignant adjacent breast tissues and found that 85 out of the 107 genes were consistently expressed in over 75% of the 294 tissues examined. Univariate analyses were used to test the association between the expression of these 85 genes with quartiles of urinary MEP using generalized linear models. Overall, four genes were significantly associated with MEP (*AREG*, *TMEM50B*, *CA8*, *C4ORF19*) regardless of menopausal status. Given the different risk factor profiles associated with pre- and postmenopausal breast cancer^36–38^ we stratified our analysis by menopausal status and found that nine and three genes were significantly associated with MEP in the pre-malignant adjacent breast tissue among postmenopausal (n = 189) and premenopausal (n = 99) women, respectively (p < 0.05, Table 2, Fig. 3A). In postmenopausal women, six genes (*AREG*, *TMC4*, *MPZL3*, *P4HA1*, *CA8*, *PLEKHA6*) had decreasing expression with increasing levels of MEP, which was consistent with results from rats where we observed reduced gene expression levels with DEP exposure. Given the limited sample size in premenopausal women, we carried out subsequent analysis in postmenopausal women only. To reduce multiple comparisons and to take advantage of the strong correlation structure among genes, a dimensionality reduction technique, i.e. factor analysis, was carried out (Fig. 4). Factor analysis revealed seven loading factors among the genes in the DEP gene signature (Table 3), of which one of the factors, factor #2, was inversely associated with urinary MEP (p < 0.05, Fig. 3B). The six genes that were identified to be inversely associated with urinary MEP in univariate analysis loaded onto factor #2. We performed transcription factor enrichment analysis of all 20 genes that loaded into factor #2 to identify putative upstream molecules that may regulate these genes. Results revealed enrichment for several transcription factor binding sites including *SOX2*, *AR*, *GATA1*, *FOXA1* and *TBX20* among these genes (p < 0.05, Table 4). Genes contributing to enrichment of *androgen receptor* (*AR*) included *SLC44A3*, *P4HA1*, *CXADR*, *AADAT*, *GALNT3* and *ABCG2*. We then restricted our analysis to *AR* + cases, which we defined as *AR* expression above the lower whisker of Tukey’s boxplot^39^ (n = 167) and found six more genes to be inversely associated with urinary MEP (Table 2).

**Table 2:** Association between DEP gene signature and MEP concentration in pre-malignant adjacent breast tissues (p < 0.05) among LIBCSP case women.

All (n = 294)	P-value	Beta	Postmenopausal (n = 189)	P-value	Beta	Postmenopausal with high AR expression (n = 167)	P-value	Beta	Premenopausal (n = 99)	P-value	Beta
*AREG*	0.014	−0.14	*AREG*	0	−0.22	*AREG*	0	−0.244	*HMGCS2*	0.006	0.41
*TMEM50B*	0.026	0.07	*TMC4*	0.008	−0.23	*SLC5A3*	0.002	0.124	*CLDN8*	0.023	0.26
*CA8*	0.03	−0.11	*TMEM50B*	0.008	0.11	*SPINT1*	0.008	−0.117	*MUC1*	0.039	0.22
*C4ORF19*	0.045	0.13	*MPZL3*	0.013	−0.17	*NAT8L*	0.01	0.262			
			*P4HA1*	0.014	−0.18	*TSPAN1*	0.011	−0.284			
			*CA8*	0.015	−0.16	*P4HA1*	0.015	−0.186			
			*SLC5A3*	0.034	0.09	*TACSTD2*	0.015	−0.137			
			*PLEKHA6*	0.039	−0.15	*TMC4*	0.016	−0.22			
			*NAT8L*	0.045	0.20	*CA8*	0.021	−0.16			
						*SLC44A4*	0.022	−0.238			
						*FXYD3*	0.023	−0.125			
						*MPZL3*	0.032	−0.153			
						*KRT18*	0.036	−0.098			
						*PLEKHA6*	0.037	−0.161			
						*TMEM50B*	0.039	0.075

**Table 3:** Factor analysis of DEP gene signature in pre-malignant adjacent breast tissues among postmenopausal women revealed 7 latent factors.

Factor	Genes loading onto factor
Factor 1	*FOXA1*,*AR*,*KRT19*,***TMEM50B***,*CBLC*,*TMEM30B*,*KRT18*,*PRSS8*,*WFDC2*,*ST6GALNAC2*,*MUC1*,***NAT8L***,*TGFB3*,*FAM134B*,*ICA1*,*HMGCS2*,*TFAP2B*
Factor 2	***P4HA1***,*RNF180*,***MPZL3***,***TMC4***,*TMEM47*,*PRLR*,***PLEKHA6***,*ACAA2*,*TOM1L1*,*GALNT3*, ***CA8***,*AADAT*,*AP1M2*,*MBOAT1*,*SLC44A3*,*CXADR*,*TNFRSF21*,*TLR2*,***AREG***,*ABCG2*
Factor 3	*RPS16*,*FXYD3*,*CD59*,*XBP1*,*SPINT2*,*IRX3*,*QSOX1*,*PTGDS*,***SLC5A3***,*TACSTD2*,*SH3YL1*,*CRABP2*,*MAL2*
Factor 4	*LCN2*,*SERPINB5*,*ANXA8L1*,*PLEKHB1*,*SLC34A2*,*PROM1*,*C4ORF19*,*CLDN8*,*SPINT1*
Factor 5	*SECTM1*,*CD14*,*PLIN2*,*CPVL*,*CD24*,*ALDOC*,*ANGPTL4*,*PPAP2C*,*ABHD14B*
Factor 6	*AQP5*,*FOLR1*,*EPCAM*,*KCNK1*,*PDZK1IP1*,*CLDN3*,*RAB25*,*ESRP1*,*FRK*,*SLC12A2*
Factor 7	*TSPAN1*,*SLC44A4*,*F13A1*
No factor assignment	*ASPN*,*CMTM8*,*PLA2G2A*,*TMEM184A*

Genes in bold are those that were significant at p < 0.05 from univariate analysis (Fig. 3), many of which load onto factor 2.

**Table 4:** Transcription factor enrichment analysis of genes in factor #2 using ChIP-X Enrichment Analysis (ChEA) database via EnrichR.

Transcription Factor	Context^a^	Species	P-value^b^	Adjusted P-value
SOX2	SW620	Human	0.000	0.187
AR	LNCAP	Human	0.003	0.340
SOX2	STOMACH	Mouse	0.011	0.519
FOXA1	ENDOMETRIOID-ADENOCARCINOMA	Human	0.020	0.564
GATA1	MEL	Mouse	0.031	0.623
AR	VCAP	Human	0.036	0.623
TBX20	HEART	Mouse	0.043	0.623
TBX20	HEART	Mouse	0.043	0.623
GATA1	MEL86	Mouse	0.043	0.623
FOXA1	PDAC	Human	0.043	0.623

^a^Refers to cell-line or tissue in which the experiment was done.

^b^The p-value is computed from a Fisher’s exact test (FET), a proportion test that assumes a binomial distribution and independence for probability of any gene belonging to any gene-set.

The top 5 transcription factors that appeared at least twice with a p-value < 0.05 are shown. ChEA is a gene-set enrichment analysis tool that tests if query gene-sets are enriched with genes that are putative targets of transcription factors, utilizing a gene-set library of transcription factors and their putative target genes curated from published ChIP-chip, ChIP-seq, and similar experiments (Lachmann *et al*. 2010).

**Figure 5:**
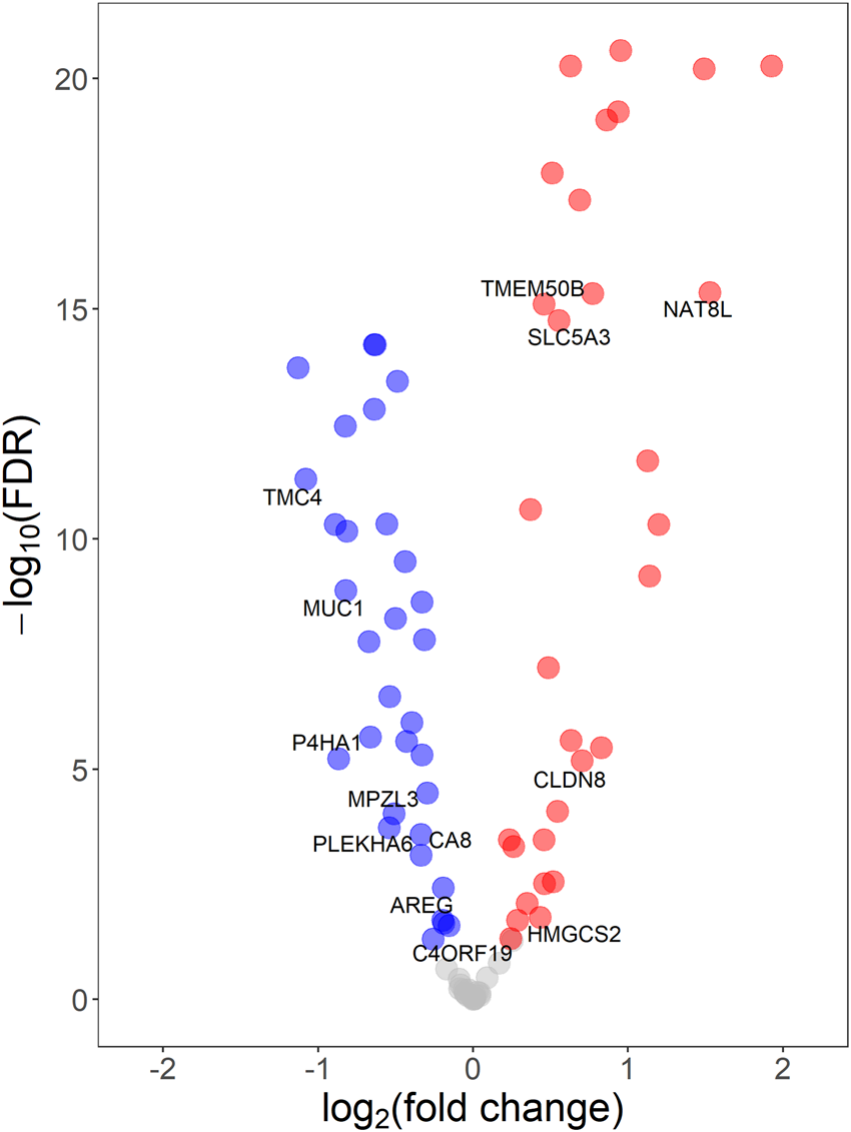
Genes significantly differentially expressed between paired tumor and adjacent breast tissues among LIBCSP postmenopausal women (FDR < 0.05). Blue indicates down-regulation and red indicates up-regulation in pre-malignant compared to tumor, respectively. Labels indicate genes found to be associated with MEP concentration (see Table 2).

We compared expression levels of 107 DEP signature genes in adjacent and their tumor counterparts (n = 243 pairs), and found that 33 genes were down-regulated and 34 genes were up-regulated in adjacent compared to corresponding tumor tissues at FDR < 0.05 (Fig. 5). In particular, all six genes found to be inversely associated with urinary MEP in adjacent tissues among postmenopausal women were also down-regulated in adjacent tissues compared to paired tumors. Results on four of these genes (*P4HA1*, *TMC4*, *MPZL3*, *PLEKHA6*) were also validated in the TCGA dataset (FDR < 0.05); however, *AREG* and *CA8* showed the opposite trend.

## Discussion

In the present study, we identified a DEP-induced gene signature using a rodent model and translated it to a population-based sample of women newly diagnosed with primary breast cancer, who had available pre-malignant adjacent and tumor tissue. To our knowledge, this is the first study of its kind to translate animal data to humans in an appropriate target tissue using a DEP dose relevant to human exposure. Rats have been widely used to study breast cancer since the pre-malignant stages of mammary cancer in rats closely recapitulate the human disease^40^.

We first identified a panel of 107 genes modified by DEP exposure in rats treated chronically from birth to adulthood using a human-level exposure that has previously been shown to result in urinary MEP concentrations within the range reported for the U.S. population^17^. By bootstrapping samples to overcome the limitation of small sample size for each experimental group (n = 5), we were able to show that even at a low, human level exposure, measurable changes in the transcriptome were detectable. The 107 modified genes in adult rats were predominantly down-regulated by DEP exposure and were enriched for biological processes such as ‘mammary gland alveolus development’. Interestingly, overexpression of at least eight genes among the DEP gene signature has been previously reported in breast cancer tissues; these include the genes *FOXA1*^41^, *PRLR*^42^, *TPH1*^43^, *AREG*^44^, *AR*^45^, *XBP1*^46^, *TGFB3*^47^ and *HMGCS2*^48^. Coordinated down-regulated expression of these genes by DEP exposure in our study seemed to suggest an inverse relationship between DEP and breast cancer in animal models. While we could not obtain such information in our animal study (all rats were sacrificed by PND 181, which is too early for spontaneous tumor formation to occur), the suggestive inverse association in our population study^13^ seems to corroborate this postulation.

We next examined the DEP gene signature in pathologically non-malignant tissues because we hypothesized that gross genomic abnormalities in tumor tissues would mask the likely subtle effects of low-dose environmental chemical exposures. In particular we identified by univariate analysis, a subset of six out of 107 genes to be negatively associated with urinary MEP in pre-malignant adjacent tissues of postmenopausal women. The down-regulated expression of these six genes with urinary MEP in humans was consistent with direction of expression change observed in rats by DEP exposure. These six genes have diverse functions. *P4HA1* encodes a component of prolyl 4-hydroxylase, a key enzyme in collagen synthesis; it was shown to be an activator of the HIF-1 pathway in breast cancer and biomarker of poor prognosis in various types of cancers including melanomas^49^, pancreatic cancer^50^ and head and neck squamous cell carcinomas^51^. *MPZL3* (Myelin protein zero like 3) is involved in mediating cell adhesion but little is known about its possible role in cancer pathways. *TMC4* encodes a transmembrane channel protein^52^; while its expression was upregulated in tumor-versus-adjacent tissue in our dataset as well as TCGA, other studies reported its downregulation in high-metastatic breast cancer cell lines compared to their low metastatic counterparts^53^ as well as breast-cancer cells compared to normal breast epithelium^54^, and its increased expression in tumors was associated with better survival of breast cancer patients^55^. *PLEKHA6* (Pleckstrin Homology Domain Containing 6) has been shown to be differentially methylated in head and neck cancer^56^; its high expression was shown to be a positive prognostic factor in lung^57^ and breast^55,58^ cancers. *CA8* (Carbonic Anhydrase 8) encodes an inhibitor of inositol trisphosphate inhibitors which regulate intracellular calcium release fundamental to many cellular processes including mitochondrial energy production and cell fate^59^. *AREG* (Amphiregulin) is a member of the epidermal growth factor family that is expressed in the mammary gland during development and has been shown to promote the growth of normal epithelial cells while inhibiting the growth of aggressive carcinoma cell lines^52^.

The strong correlation structure underlying genes in adjacent tissues enabled us to use factor analysis to derive seven factors, clusters of highly correlated genes, of which one factor, factor 2, showed independent negative association with urinary MEP. Interestingly, all six genes identified by univariate analysis loaded into factor #2, indicating similar expression patterns of these genes. Furthermore, we showed that the six genes that were negatively associated with urinary MEP were also coordinately down-regulated in adjacent compared to paired tumor tissue, and four out of six of these genes were also down-regulated in adjacent compared to paired tumors of the TCGA dataset. Collectively, our results from humans and animals seem to suggest an inverse relationship between DEP exposure and breast cancer development. However, we acknowledge the cross-sectional nature of our study, where urinary MEP concentrations were obtained only at one time-point after breast cancer diagnosis, and may not necessarily reflect lifetime exposure to DEP or whether the exposure occurred prior to diagnosis of breast cancer. Additionally, there is no control (cancer-free) population in the study. Hence, whether phthalate-induced transcriptome changes modify breast cancer susceptibility needs to be further investigated to clarify the etiologic mechanism of phthalates in breast carcinogenesis. Design of the current study does not allow us to claim the direct link between DEP exposure and breast cancer risk. We also have to acknowledge a seeming discrepancy of our results with the studies of López-Carrillo *et al*.^10^ (higher urine levels of MEP associated with increased BC risk) and Sprague *et al*.^11^ (elevated serum levels of MEP associated with increased breast density) for which we currently don’t have an explanation.

To gain insight about upstream molecular regulators of genes associated with urinary MEP in postmenopausal tumor-adjacent breast tissues, we performed transcription factor enrichment analysis of the 20 genes belonging to factor 2 and observed enrichment for AR binding sites among these genes. The anti-androgenic activity of phthalates has been extensively reported, which include endpoints such as shorter anogenital distance and reduced testosterone levels^5,60–62^. While the mechanism of anti-androgenic activity of phthalates has been shown to be mediated by inhibition of testosterone synthesis leading to Leydig cell dysfunction^62,63^, several phthalates have been shown to be able to bind to AR weakly suggesting that phthalates may elicit their effects through both receptor- and non-receptor-mediated pathways^64^. Even though in our study *AR* was among the signature panel of genes disrupted by DEP in rats, its expression was not significantly altered with urinary MEP exposure in human tissues. However, we noted that among postmenopausal women with high *AR* expression, six more genes were significantly associated with urinary MEP, suggesting that *AR* could play a role in the association between phthalate exposure and gene expression.

Our results indicated stronger associations of DEP signature genes among adjacent tissues of postmenopausal women, and not premenopausal women; part of this could be attributed to the smaller sample size among premenopausal (n = 99) compared to postmenopausal (n = 189) women. Premenopausal breast cancers tend to be more aggressive^36^, be associated with poorer survival^37^ and have distinct molecular profiles compared to postmenopausal breast cancers^38^. There was no major difference in distribution of MEP concentrations between premenopausal and postmenopausal women in our dataset. Whether there are different environmental etiologies of premenopausal compared to postmenopausal breast cancers remains to be elucidated.

We found a larger number of genes modified among parous rats (339 genes) compared to nulliparous rats (43 genes) suggesting that parity may play a role in modifying the effects of phthalate exposure on the mammary gland transcriptome. The parous rats in our study completed pregnancy and lactation and were sacrificed 35 days after the end of lactation. Post-lactational involution occurs within a few days of end of lactation whereby apoptosis of secretory structures remodels the gland to its pre-pregnant ductal architecture resembling its virgin counterpart^65^. That we did not observe significant gene expression changes between parous and nulliparous rats suggests that the parous rats in our study had finished involution. Parity is known to reduce the risk of developing breast cancer^66^; however how environmental factors interact with parity to influence cancer susceptibility is unknown. Our results suggesting that phthalate exposure had a more profound effect on the transcriptome of parous compared to nulliparous rats warrants further investigation, for example, by examining rates of spontaneous mammary tumor formation among exposed and control groups later in life.

We acknowledge some limitations of our study. First is the translation of findings from rats to humans. While rats have been a useful model system to study breast cancer, important species-specific differences exist, including an accelerated lifespan leading to condensed mammary developmental timing in rats, six pairs of mammary glands in rats compared to one in humans^67^ as well as species-specific differences in response to environmental chemicals^68^. Nevertheless, Sprague-Dawley rats have been shown to be one of the most physiologically relevant and genetically defined animal models for studying human sporadic breast cancer^69,70^. For example, they share similar age-equivalent distribution of mammary tumors as in human population; mammary carcinomas from this animal model also share many morphological and molecular features, including estrogen-dependence, chromosomal instability, aneuploidy, and deregulation of cell cycle with breast cancer in human populations^71^. Additionally, we did not have a technical possibility to run additional assays such as *in situ* hybridization experiments or immunohistochemical staining to confirm the expression changes in the caudal mammary glands. Second is the translation of findings from normal mammary glands in rats to cancer-adjacent breast tissues in humans. Extratumoral benign-appearing tissues lying adjacent to breast tumors have been shown to harbor genomic alterations such as somatic loss of heterozygosity, methylation changes and shortened telomeres, and in some cases may have been infiltrated with tumor cells^72^ – the so-called “field effect” – making it harder to untangle the subtle effects of low-dose environmental chemical exposures on transcriptome pattern. Finally, the route of exposure used in animal studies involved oral gavage, while exposures to many environmental chemicals, including phthalates have been shown to occur via multiple routes other than oral, such as dermal or by inhalation^62^. However, we attempted to resolve this issue by calibrating the exposure dose to achieve similar urinary biomarker concentration in rats and humans.

## Conclusion

Using an integrative and translational approach, we identified a DEP gene signature in an animal model which was partially validated in a human population. Functional relevance of the gene signature indicated by in silico pathway analysis further support the potential causal role of phthalate in breast carcinogenesis.

## Supplementary information

Table S1.

## Data Availability

Microarray data have been deposited in NCBI’s Gene Expression Omnibus (GEO) and are accessible through GEO Series accession number GSE95554.
