# Supplementary material for: Gene expression profiles for low-dose exposure to diethyl phthalate in rodents and humans: a translational study with implications for breast carcinogenesis
Source: Sci Rep. 2020 Apr 27;10:7067. doi: 10.1038/s41598-020-63904-w (PMC7184607; doi:10.1038/s41598-020-63904-w)
Supplement: Supplementary file 1 — Table S1. [file 41598_2020_63904_MOESM1_ESM.docx]

**Supplementary Information** for the paper:

**Gene expression profiles for low-dose exposure to diethyl phthalate in rodents and humans: a translational study with implications for breast carcinogenesis**

Kalpana Gopalakrishnan^1*^, Vasily N. Aushev^1*^, Fabiana Manservisi^2^, Laura Falcioni^2^, Simona Panzacchi^2^, Fiorella Belpoggi^2^, Humberto Parada Jr^3^, Gail Garbowski^4^, Hanina Hibshoosh^5^, Regina M. Santella^4^, Marilie D. Gammon^6^, Susan L. Teitelbaum^1^, Jia Chen^1,7,8,9,@^

**Table S1. DEP gene signature identified in rats.** Samples were bootstrapped to choose 4 out of 5 samples in DEP and in control groups, resulting in 25 rounds of differential gene expression analysis each for parous and for nulliparous groups carried out by limma using a FDR < 0.25 and fold change ≥ 1.5. ‘+’ indicates that the gene is present and ‘-‘ indicates that the gene is absent.

| Gene symbol | DEP  parous signature | DEP  nulliparous signature |
| --- | --- | --- |
| *AADAT* | + | - |
| *ABCG2* | + | - |
| *ABHD14B* | + | - |
| *ACAA2* | - | + |
| *ADGRE1* | - | + |
| *ALDOC* | + | + |
| *ANGPTL4* | + | - |
| *ANKRD22* | + | - |
| *ANXA8L1* | + | - |
| *AP1M2* | + | - |
| *AQP5* | + | - |
| *AR* | + | - |
| *AREG* | + | - |
| *ARG2* | + | - |
| *ASPN* | + | - |
| *BCL2L15* | + | - |
| *C4ORF19* | + | + |
| *CA8* | + | - |
| *CBLC* | + | + |
| *CCL15-CCL14* | + | - |
| *CD14* | + | - |
| *CD24* | + | + |
| *CD59* | + | - |
| *CLDN3* | - | + |
| *CLDN8* | + | + |
| *CMTM8* | + | - |
| *CPVL* | + | - |
| *CRABP2* | + | - |
| *CTH* | + | - |
| *CXADR* | + | - |
| *DUOXA1* | + | + |
| *ENPP3* | + | - |
| *EPCAM* | + | - |
| *ESRP1* | + | - |
| *F13A1* | + | - |
| *FAM110C* | - | + |
| *FAM134B* | + | + |
| *FGG* | + | - |
| *FOLR1* | + | - |
| *FOXA1* | + | - |
| *FRK* | + | - |
| *FXYD3* | + | - |
| *GALNT3* | + | - |
| *HEPACAM2* | + | - |
| *HMGCS2* | + | - |
| *ICA1* | + | - |
| *IGSF5* | + | - |
| *IRX3* | + | - |
| *KCNK1* | + | + |
| *KRT18* | + | - |
| *KRT19* | + | - |
| *LBP* | + | - |
| *LCN2* | + | - |
| *MAL2* | + | + |
| *MBOAT1* | + | + |
| *MPZL3* | - | + |
| *MUC1* | + | - |
| *MUC15* | + | - |
| *NAT8L* | - | + |
| *ORM1* | + | - |
| *P4HA1* | - | + |
| *PDZK1IP1* | + | - |
| *PLA2G2A* | + | - |
| *PLEKHA6* | + | - |
| *PLEKHB1* | - | + |
| *PLET1* | + | + |
| *PLIN2* | + | - |
| *POF1B* | + | - |
| *PPAP2C* | + | - |
| *PRLR* | + | - |
| *PROM1* | + | - |
| *PRSS8* | + | - |
| *PTGDS* | + | - |
| *QSOX1* | + | - |
| *RAB25* | + | + |
| *RNF128* | + | + |
| *RNF180* | + | - |
| *RPS16* | - | + |
| *SCNN1B* | + | - |
| *SDR16C5* | + | - |
| *SECTM1* | + | + |
| *SERPINB5* | + | - |
| *SH3YL1* | + | - |
| *SLC12A2* | + | - |
| *SLC34A2* | + | - |
| *SLC44A3* | + | - |
| *SLC44A4* | + | + |
| *SLC5A3* | - | + |
| *SOWAHB* | - | + |
| *SPINT1* | - | + |
| *SPINT2* | - | + |
| *ST6GALNAC2* | + | - |
| *TACSTD2* | + | - |
| *TFAP2B* | + | - |
| *TGFB3* | + | - |
| *TLR2* | - | + |
| *TMC4* | + | - |
| *TMEM184A* | + | - |
| *TMEM30B* | + | - |
| *TMEM47* | - | + |
| *TMEM50B* | - | + |
| *TNFRSF21* | + | + |
| *TOM1L1* | + | - |
| *TPH1* | + | - |
| *TSPAN1* | + | - |
| *WFDC2* | + | + |
| *XBP1* | + | + |
